# Optimized New Shengmai Powder modulation of cAMP/Rap1A signaling pathway attenuates myocardial fibrosis in heart failure

**DOI:** 10.1186/s13020-024-00902-4

**Published:** 2024-02-24

**Authors:** Zeyu Zhang, Zhe xu, Shuai Wang, Zhuangzhuang Jia, Zhou zhou, Ci Wang, Shanshan Lin, Yiting Feng, Xianliang Wang, Jingyuan Mao

**Affiliations:** 1 First Teaching Hospital of Tianjin University of Traditional Chinese Medicine, National Clinical Research Center for Chinese Medicine Acupuncture and Moxibustion, Tianjin, 300381 People’s Republic of China; 2 Tianjin University of Traditional Chinese Medicine, Tianjin, 301617 China

**Keywords:** Optimized New Shengmai Powder, cAMP/Rap1A signaling pathway, Myocardial fibrosis, Heart failure, Traditional Chinese medicine

## Abstract

**Background:**

Optimized New Shengmai Powder (ONSMP) is a traditional Chinese medicine formula with significant anti-heart failure and myocardial fibrosis effects, but the specific molecular biological mechanisms are not fully understood.

**Methods:**

In this study, we first used network pharmacology to analyze the ONSMP's active ingredients, core signaling pathways, and core targets. Second, calculate the affinity and binding modes of the ONSMP components to the core targets using molecular docking. Finally, the heart failure rat model was established by ligating the left anterior descending branch of the coronary artery and assessing the effect of ONSMP on myocardial fibrosis in heart failure using echocardiography, cardiac organ coefficients, heart failure markers, and pathological sections after 4 weeks of drug intervention. The cAMP level in rat myocardium was determined using Elisa, the α-SMA and FSP-1 positive expression determined by immunohistochemistry, and the protein and mRNA levels of the cAMP/Rap1A signaling pathway were detected by Western Blotting and quantitative real-time PCR, respectively.

**Results:**

The result shows that the possible mechanism of ONSMP in reducing myocardial fibrosis also includes the use of 12 active ingredients such as baicalin, vitamin D, resveratrol, tanshinone IIA, emodin, 15,16-dihydrotanshinone-i to regulate β1-AR, AC6, EPAC1, Rap1 A, STAT3, and CCND1 on the cAMP/Rap1A signaling pathway, thereby inhibiting the proliferation of cardiac fibroblasts and reduce the excessive secretion of collagen, effectively improve cardiac function and ventricular remodeling in heart failure rats.

**Conclusion:**

This research shows that ONSMP can inhibit myocardial fibrosis and delay heart failure through the cAMP/Rap1A signaling pathway.

**Supplementary Information:**

The online version contains supplementary material available at 10.1186/s13020-024-00902-4.

## Introduction

Heart failure is an end-stage cardiovascular disease in which the persistent stimulation of various factors causes structural defects in the myocardium and limitations in cardiac function [1]. In China, the prevalence of heart failure among people aged 35–74 years was 0.9% in 2000 [2], and the majority of heart failure among people aged 35 years or older was 1.3% in 2012–2015 [3], indicating that the overall prevalence of heart failure in China has increased by 44% during the 15 years at the beginning of this century. The 2020 China Heart Failure Medical Quality Control Report showed that the mortality rate of hospitalized patients with heart failure in China was 2.8% [4]; an epidemiological survey in Beijing showed that the 5-year all-cause mortality rate of heart failure patients was 55.4%, the cardiovascular mortality rate was 49.6%, and the median survival time was only 34 months [5]. Therefore, the prevention and treatment of heart failure is an urgent scientific challenge. Myocardial fibrosis is an essential pathological change in the whole process of heart failure caused by excessive deposition, imbalance, and disarrangement of various collagen proteins in the extracellular matrix of the heart, which can irreversibly limit the heart’s pumping function [6]. Intervention in myocardial fibrosis is a top priority in preventing and treating heart failure, and the relevant regulatory pathways have significant research value and clinical translational significance.

Chinese medicine treatment of heart failure has the synergistic effect of further improving patients' clinical manifestations, improving quality of life, increasing exercise tolerance, and even improving prognosis. Combining Chinese and Western medicine has become an objective and widely recognized diagnostic and therapeutic model in the clinical practice of heart failure treatment in China [7]. Based on the clinical manifestations of fatigue, respiratory distress, edema, and blood stasis in patients with heart failure, Chinese medicine summarizes the pathomechanism as asthenia in origin and asthenia in superficiality, with the interaction of cardiac qi, blood, yin, and yang deficiency and the pathological products of blood stasis, phlegm, and dampness as the main factors [8]. The earliest Chinese medical classic, Huangdi’s classic on medicine, has the record “heart controlling blood circulation” that the heart gas and can promote the bloodline; the bloodline is smooth then the whole body nourishes the organs. Given this, the nationally famous Chinese medicine, Qihuang scholar Professor Mao Jingyuan set many years of clinical experience and research concluded Optimized New Shengmai Powder (ONSMP), which has benefit Qi, invigorate blood circulation, and diuretics efficacy. The herbs in ONSMP combination synergistically inhibit the development of heart failure, such as the Sovereign drug *Astragalus membranaceus* (Fisch.) Bung 10 *g*, which can replenish vitality to solid consolidate, and tonify without causing stagnation and has the function of acting Qi to reduce dampness; Ministerial drugs *Codonopsis pilosula* (Franch.), *Eleutherococcus senticosus* (Rupr. & Maxim.) Maxim., and *Salvia miltiorrhiza* Bunge all have 10 *g*, which can invigorate qi to channel the veins; Adjuvant drugs *Trionyx sinensis Wiegmann* and *Poria cocos* (Schw.) Wolf both have 10 *g*, can activate blood and water; *Lepidium apetalum* Willd has 6 *g*, can disperse bitter diarrhea, cold decrease, and flow lung qi to facilitate control and regulate human blood circulation; *Citrus* × *aurantium* L. has 6 *g* and can assist all medicines to promote qi; *Ophiopogon japonicus* (Thunb.) Ker Gawl. has 10 *g* that can nourish blood and rejuvenate veins to prevent injury to yin [9]. Clinical studies have shown that ONSMP has good efficacy and safety in treating heart failure, which is conducive to improving patients' cardiac function and can further improve their exercise tolerance and quality of life [10, 11]. Through experimental studies, Pang Lei et al. [12]. Confirmed that ONSMP has a significant cardioprotective effect, which can significantly increase the survival rate and improve the weight-bearing exhaustion swimming time of mice with adriamycin-induced myocardial injury; Zhang Zeyu et al. [9] demonstrated that ONSMP can attenuate myocardial fibrosis by inhibiting the activation of the MAPK signaling pathway; Wang Shuai et al. [13] showed that ONSMP can improve cardiac function and exercise tolerance in heart failure rats by regulating the ubiquitin–proteasome signaling pathway; Hou Yazhu et al. [14] confirmed that ONSMP could inhibit cardiomyocyte apoptosis in heart failure rats by regulating the NMDAR signaling pathway.

Given the multi-component, multi-pathway, and multi-target nature of TCM, fully deciphering its potential mechanism of action remains a formidable challenge. This study continued the previous work, analyzed the mechanism of ONSMP regulating heart failure and myocardial fibrosis from macro to micro levels and from multiple perspectives with the help of network pharmacology methods, and predicted the binding activities and binding forms of ONSMP active components and core targets using molecular docking technology. Finally, ligation of the left anterior descending branch of the coronary artery to construct a rat model of heart failure for in vivo experimental validation of the signaling pathways and targets screened by Network Pharmacology to reveal the possible biological mechanisms of ONSMP to improve myocardial fibrosis in heart failure at multiple levels, from efficacy to molecular mechanism, from serum to tissue, and from proteins to genes, to provide an exhaustive theoretical basis for the clinical use of ONSMP.

## Materials and methods

### Screening ONSMP active ingredient targets and disease proteins

Firstly, all ingredients were obtained from the HERB database (http://herb.ac.cn/) using the names of the nine ONSMP herbs as search terms. We utilized the PubChem database (https://pubchem.ncbi.nlm.nih.gov/) to query the composition of canonical smiles of the ONSMP components and subsequently imported it into the SwissADME database (http://www.swissadme.ch/). Based on the screening criteria shown in Table 1, we selected ONSMP active ingredients with good oral bioavailability and pharmacokinetic properties. After that, use the HERB database (http://herb.ac.cn/), SwissTargetPrediction database (http://old.swisstargetprediction.ch/), Similarity ensemble approach database (https://sea.bkslab.org/), and Super-PRED database (https://prediction.charite.de/index.php) to obtain the targets of ONSMP active ingredients. The disease proteins were obtained from HERB database (http://herb.ac.cn/), OMIM database (https://omim.org/), GeneCards database (http://www.genecards.org/), PharmGkb database (http://www.pharmgkb.org/), TTD database (http://db.idrblab.net/ttd), and DrugBank database (https://www.drugbank.ca/) with “chronic heart failure” and “myocardial fibrosis” as the search terms.

**Table 1:** Screening conditions of ONSMP active ingredients

Filter conditions	Category	Class
Water Solubility	Log S (ESOL)	Soluble or moderately soluble or very soluble
	Log S (Ali)	
	Log S (SILICOS-IT)	
Pharmacokinetics	GI absorption	High
Druglikeness	Lipinski	Yes; 0 violation
	Ghose	Yes
	Veber	Yes
	Egan	Yes
	Muegge	Yes

### Screening pathways and core targets of ONSMP on myocardial fibrosis in heart failure

We first installed the Bioconductor data package (including DOSE, clusterProfiler, enrichplot, and pathview) in R programming language 4.2.0 software, after which we imported the intersection proteins of heart failure proteins, myocardial fibrosis proteins, and ONSMP active ingredients target proteins were imported into this software to perform GO and KEGG enrichment analyses with a *P*-value of less than 0.05 as the screening condition. Obtain key signaling pathways and corresponding proteins of ONSMP for the treatment of myocardial fibrosis in heart failure from the results of KEGG enrichment analysis, then import these proteins into the string database (https://string-db.org/), with the settings of species as homo sapiens, confidence level ≥ 0.4, and hidden discontinuous nodes, to obtain the interactions of these proteins. Use Cytoscape-v3.9.0 software to calculate the betweenness centrality, closeness centrality, degree centrality, eigenvector centrality, local average connectivity, and network centrality of the prediction results of the string database and retain the proteins with these parameters greater than their respective mean values. After screening twice, the remaining nodes were the core proteins. Then, construct component-protein networks for these core proteins with the corresponding ONSMP active ingredients and calculate the degree of centrality by Cytoscape-v3.9.0 software.

### Molecular docking of ONSMP active ingredients to pathway core targets

The molecular docking approach examines the ability of core proteins to interact with the corresponding ONSMP components and predicts the binding mode and affinity between them. Receptor proteins and ligand compounds were first obtained and processed: jointly using the Uniport database (https://www.uniprot.org/) and the AlphaFold Protein Structure Database (https://www.alphafold.ebi.ac.uk/) to acquire the receptor proteins β1-AR (Uniport ID: P08588), AC6 (Uniport ID: O43306), EPAC1 (Uniport ID: O95398), RAP1A (Uniport ID: P62834), STAT3 (Uniport ID: P40763), and CCND1 (Uniport ID: P24385) 3D structure files, and then use PyMOL 2.3.0 software to check the protein structures and remove water molecules, irrelevant protein chains and proto-ligands in the protein structure for docking; using the Pubchem database (https://pubchem.ncbi.nlm.nih.gov/) to acquire the ligand compounds baicalin (Pubchem CID: 64982), vitamin D (Pubchem CID: 12895043), resveratrol (Pubchem CID: 445154), Tanshinone IIA (Pubchem CID: 164676), apigenin (Pubchem CID: 5280443), calycosin (Pubchem CID: 5280448), capsaicin (Pubchem CID: 1548943), cryptotanshinone (Pubchem CID: 160254), emodin (Pubchem CID: 3220), rhein (Pubchem CID: 10168), ursolic acid (Pubchem CID: 64945), 15,16-dihydrotanshinone i (Pubchem CID: 11425923) 3D structure files, and then use Chem3D 2020 software to optimize the molecular force field of the ligand compound structure to finally obtain the optimal molecular structure in the lowest energy state. Molecular docking of receptor proteins and ligand compounds: Hydrogenation of proteins and hydrogenation and determination of torsion bonds of ligand compounds were performed using AutoDock Tools 1.5.6 and saved as pdbqt format files. Use the grid plate to set up molecular docking parameters, as shown in Table 2. Set the docking method as semi-flexible-docking and the docking algorithm as Lamarckian genetic algorithm. Run Auto Dock Vina1.2.0 for molecular docking to get the docking binding free energy as well as the docking result file, and then convert the PDBQT format of the complexes to PDB format by Open Babel GUI software, and then import them into PyMOL2.3.0 software and Discovery Studio2019 software for 3D and 2D transformation respectively.

**Table 2:** Molecular docking parameters

Protein	Center_x	Center_y	Center_z	Size_x	Size_y	Size_z
β1-AR	− 65.454	22.618	− 6.889	86.1	68.3	79.3
AC6	− 9.037	− 1.3	3.443	126	100	116
Epac1	8.839	− 4.544	− 6.889	112	100	116
Rap1A	8.839	21.511	24.47	60	58	40
STAT3	6.788	25.175	18.319	60	32.25	47.25
CCND1	13.701	8.901	37.837	26.25	30	30

### Drugs preparation

The Pharmacy Department of the First Affiliated Hospital of Tianjin University of Traditional Chinese Medicine provided all the herbs. Grind Salvia miltiorrhiza Bunge into powder, while *Astragalus membranaceus* (Fisch.) Bunge, *Codonopsis pilosula* (Franch.) Nannf, *Eleutherococcus senticosus* (Rupr. & Maxim.) Maxim., *Salvia miltiorrhiza* Bunge, *Trionyx sinensis* Wiegmann, *Poria cocos* (Schw.) Wolf, *Lepidium apetalum* Willd., *Ophiopogon japonicus* (Thunb.) Ker Gawl., and *Citrus* × *aurantium* L. were decocted with ten times the amount of water for 2 h and then filtered. Decocted dregs of the herbs with another ten times the amount of water for 2 h and then filtered. The two filtrates were combined, concentrated, and dried to make a powder and mixed with the powder of *Salvia miltiorrhiza* Bunge. 82 *g* ONSMP raw drug processed to obtain patent medicine is 31 *g*. We have examined the components contained in ONSMP by UHPLC-Q-Exactive-MS/MS in a previous study [9]. The positive drug of choice was the ACEI representative drug captopril Captopril (CAP, H31020564, Shanghai Xudong Haipu Pharmaceutical Co., Ltd., China), which inhibits ventricular remodeling by reducing Ang II production and has an activating effect on the β1-AR-cAMP signaling pathway [15–17].

### Animals

All animal experimental procedures were performed by the National Institutes of Health Guidelines for Laboratory Animals (NIH Publications No. 8023, revised 1978) and approved by the Experimental Animal Ethics Committee of the Institute of Radiological Medicine, Chinese Academy of Medical Sciences. 55 healthy male SD rats were purchased from Beijing Viton Lihua Laboratory Animal Technology Co, Ltd, with animal production license No. SCXK (Beijing) 2021-0006. We housed rats at the Institute of Radiology, Chinese Academy of Medical Sciences, at a temperature of (22 ± 2) ℃ and a humidity of (40 ± 5) %, with a 12 h light–dark cycle and free access to water. Rats were divided into sham (n = 9) and operation (n = 46) groups, while later anesthetized using intraperitoneal injection of 1.5% tribromoethanol, tracheal intubated, and connected to a small animal ventilator (tidal volume of 6 ml, respiratory rate of 80 breaths/min, respiratory ratio 1:2). Two professionals opened the thoracic cavity at the 3rd and 4th intercostal spaces of rats in the operation group to expose the heart, then ligated the left anterior descending branch of the coronary artery with a 5–0 suture 2 mm below the left atrial appendage. Finally, sutured the thoracic cavity of the rats one layer at a time with a 2–0 suture and turned off the ventilator. Sham group rats underwent thoracotomy without ligation of the left anterior descending coronary artery, and the rest of the treatment was the same as above. Penicillin 800,000 units was injected intraperitoneally for three consecutive days to prevent infection. At the end of the 8th week after surgery, a total of 17 rats in the operation group died (the mortality rate was 37%), and no deaths occurred in the sham group. Then, using LVEF ≤ 50% as the inclusion criterion [18], 24 rats with heart failure were screened from the 29 rats surviving in the operation group and randomly divided into the model, ONSMP-L, ONSMP-H, and CAP groups (6 rats in each group). Based on the conversion formula for the body surface area of humans and rats, we derived that the equivalent drug dose in rats was 6.3 times that in humans. Thus, 6.3 times the ONSMP dose in humans is 2.79 *g*/kg, which we used for the gavage dose of rats in the ONSMP-L group, and later set the drug concentration of ONSMP-H to 2 times that of ONSMP-L (5.58 *g*/kg); the positive drug CAP gavage dose was 2.25 mg/kg. At the same time, six rats were randomly selected in the sham group and gavaged with saline along with the model group rats. The frequency of gavage in rats was once a day for 4 weeks.

### Cardiac function, organ coefficients, and Elisa assay

At the end of the gavage, we measured the LVEF, LVFE, IVSd, IVSs, LVIDd, LVIDs, LVPWd, and LVPWs of the rats by ultrasound. At the end of the experiment, 1.5% tribromoethanol was injected intraperitoneally into the anesthetized rats. Then, open the abdominal cavity of the rats and collect blood from the abdominal aorta with a sterile blood collection needle and procoagulant tube. Removed the heart and right-leg tibia, washed with normal saline, dried with filter paper, weighed, and calculated HWI and HW/TL. After separating the right heart, weighing the left ventricular weight, and measuring the length of the short and long axis of the left ventricle, we calculate LVWI, LVW/TL, and SI. The blood of the rats was left at room temperature for 30 min and then centrifuged at 3500 rpm (10-cm radius) for 15 min, and the serum was collected; 50 mg of myocardium was homogenized by mixing it with 450 ml of PBS and then centrifuged at 5000 rpm (10 cm radius) for 15 min to collect the supernatant. The levels of ANP, BNP, NT-ProBNP, PICP, MMP-2, MMP-9, TIMP-1, and cAMP were detected by an enzyme-linked immunosorbent kit (ANP: MM-21097R1, BNP: MM-0067R1, NT-ProBNP: MM-0329R1, PICP: MM-0621R1, MMP-2: MM-0111R1, MMP-9: MM-20918R1, TIMP-1: MM-0056R1; cAMP: MM-0549R1, Jiangsu Meiman Industrial Co., Ltd.) using a multifunctional enzyme labeling instrument (MULTISKANFC. Thermo Scientific, Massachusetts, USA) to analyze the absorbance of the test wells and calculate the corresponding index levels.

### Histopathological staining and immunohistochemistry

Myocardial tissues from the infarcted area of rats were immersed in 4% paraformaldehyde for 3 days, paraffin-embedded, and cut into 4 or 6 μm sections. In contrast, the myocardium was later stained for histopathological examination using Hematoxylin–eosin (HE), Sirius red, and Masson staining kits (HE: BP-DL001-100 mL, Sirius red: BP-DL030-500 mL, Masson: BP-DL022-50 mL, Nanjing Sempega Biotechnology Co., Ltd., Nanjing, China) for histopathological examination of the myocardium. For HE staining, 4 μm sections in an electrically heated blast drying oven at 60 ℃ for 3 h, anhydrous ethanol immersion twice (5 min/time), 75% ethanol immersion for 5 min, distilled water rinsing for 5 min, hematoxylin solution staining for 5 min, distilled water rinsing for 5 min, 1% hydrochloric acid alcohol wash for 30 s, distilled water wash for 15 min, 0.5% Eosin staining solution for 2 min, and distilled water wash for 30 s, 80% ethanol wash for 30 s, 95% ethanol immersion twice (1 min/time), anhydrous ethanol immersion twice (3 min/time), xylene immersion twice (3 min/time), and neutral gum sealing. For Sirius red staining, 6 μm sections were dewaxed to water, then immersed in Sirius red staining solution for 60 min, rinsed in distilled water for 30 s, rinsed in weak acidic working solution for 1 min, immersed in Mayer’s hematoxylin staining solution for 10 min, rinsed in distilled water for 10 min, dehydrated, transparent, and sealed. For Masson staining, 4 μm sections were dewaxed to water, stained with an equal mixture of Weigert hematoxylin A and Weigert hematoxylin B for 10 min, washed in water for 1 min, and then stained with Masson's complex staining solution for 15 min, douche briefly in distilled water, and treated with 1% phosphomolybdic acid solution for 5 min, aniline blue staining solution for 5 min, and 1% glacial acetic acid in water for 1 min, dehydrated, transparent and sealed.

Subsequently, a universal immunohistochemistry kit (WE0316, Beijing Boosun Biotechnology Co, Ltd, Beijing, China) detected positive expression of α-SMA and FSP-1. The 4 μm sections were dewaxed and rinsed 3 times (5 min/time) with PBS, added 3% H2O2 (80% methanol) dropwise, and left at room temperature for 10 min before being rinsed again with PBS and cooled to room temperature. Placed section in hot Sodium Citrate Antigenic Repair Solution for 10 min for hot antigen repair, cooled to room temperature, and rinsed 3 times (5 min/time) with PBS, later immersed in goat serum sealing solution for 20 min, followed by a drop of 50 μL of antibody and kept at 4 ℃ for 12 h before being rinsed again with PBS. The antibodies α-SMA (ab124964) and FSP-1 (ab220213) were purchased from Abcam (Cambridge, UK).

The sections for HE, Sirius red, Masson, and immunohistochemistry were magnified 400 times on an orthogonal light microscope (Nikon Eclipse E100, Nikon Corp., Tokyo, Japan) with 3 random fields of view, and then the CVF of Sirius red and Masson stained sections and the α-SMA and FSP-1 positive expression of immunohistochemistry sections were calculated by using Image J image analysis software.

### Western blotting

Protein samples were electrophoresed on a 10% electrophoresis gel and then transferred to PVDF membranes, immersed in rapid containment solution for 30 min, then washed three times with 1 × TBST (10 min/wash), and engaged in polyclonal primary antibody for 12 h at 4 ℃. The antibodies β1-AR (ab85037), AC6 (ab14781), Epac1 (ab124162), STAT3 (ab68153), p-STAT3 (ab76315), CCND1 (ab16663), α-SMA (ab124964), FSP-1 (ab220213), and GAPDH (ab9485) purchased from Abcam (Cambridge, UK); Rap1A (DF6157) purchased from Affinity Biosciences (Jiangsu, China). Washed PVDF membrane with 1 × TBST three times (10 min/time), immersed with a secondary antibody for 2 h and washed with 1 × TBST three times (10 min/time). The strips were immersed in ECL developer for 1 min, then detected by a multifunctional imager (97-0827-02, Jena Analytics, Germany), and analyzed for grayscale values with Image J image analysis software.

Assayed Rap1A activity levels were according to the kit instructions (Abcam, ab212011, Cambridge, UK). Briefly, 50 mg of myocardial tissue was clipped from the rat apical infarct site and washed with ice-cold PBS, then 1 ml of 1 × analytical buffer was added and homogenized. The supernatant was collected by centrifugation in a high-speed cryo-centrifuge at 14,000 × *g* for 10 min at 4 ℃. After that, incubate the supernatant for 30 min with agarose beads coupled to the Rap‐binding domain of RalGDS, which binds specifically to the active form of Rap1. Subsequently, the precipitated GTP‐Rap1A was detected by Western blot analysis using anti‐Rap1A antibody, and analyzed for grayscale values with Image J image analysis software.

### qPCR

Incorporate 1 mL of RNA extract (G3013, Servicebio, Wuhan, China), 20 mg of myocardial tissue, and grinding beads into a grinding tube, and subject to thorough grinding using a three-dimensional freeze grinder. At 4 ℃, centrifuge at 12,000 rpm for 10 min. Take the supernatant and add 400 μL of chloroform, mix well, and let stand for 3 min. Then, centrifuge again at 12,000 rpm for 10 min at 4 ℃, and transfer 400 μL of supernatant to a new centrifuge tube. Add 550 μL of isopropanol to the supernatant mix well and let stand for it at – 20 ℃ for 15 min. Centrifuge it at 12,000 rpm for 10 min at 4 ℃. The white sediment at the bottom of the tube is the RNA. After removing the liquid, add 1 mL of 75% ethanol and mix by inverting the tube to wash the sediment. The centrifuge tube will be centrifuged at 4 ℃ at 12,000 rpm for 5 min and placed on a super-clean table for 3 min after removing the supernatant. Then, 15 μL of RNA solubilizing solution was added and incubated at 55 ℃ for 5 min. The RNA concentration and purity were measured using an ultra microspectrophotometer (NanoDrop2000, Thermo Scientific, Massachusetts, USA). Using the reverse transcription reagent kit (G3337, Servicebio, Wuhan, China) to prepare the reverse transcription reaction system, gently mix, then centrifuge. Processed reaction mixture was on the PCR instrument (DS-11, Thermo Scientific, Massachusetts, USA) with the program set to 25 ℃ for 5 min, 42 ℃ for 30 min, and 85 ℃ for 5 s. Take 0.1 mL of the PCR reaction plate to establish the reaction system, and set the PCR amplification program on the fluorescence quantitative PCR instrument (CFX Connect, Bio-rad, California, USA): 95 ℃ for 30 s, 95 ℃ for 15 s and 60 ℃ for 30 s (40 cycles), with fluorescence signals collected at 0.5°C per temperature rise from 65 ℃ to 95 ℃. The sequences of the primer sets used for this analysis were as follows (Table 3). Use the 2^−ΔΔ^Ct method to quantify each gene's relative expression level.

**Table 3:** Primers’ sequences used in this study

Gene		Sequences of primers
*β1-AR*	F	5ʹ-CGCTGCTACAACGACCCCAAG-3ʹ
	R	5ʹ-CGGAGGTACACGAAGGCCATG-3ʹ
*AC6*	F	5ʹ-AATGTCAGCATCCTGTTTGC-3ʹ
	R	5ʹ-AGGTCATGACCAGTTCCTG-3ʹ
*Epac1*	F	5ʹ-CCAGGTGAGAACCACTGGCA-3ʹ
	R	5′-CGAACACTAGCTGGTAAGAGCA-3ʹ
*Rap1A*	F	5ʹ-GTCCACACACCTGGCAAATC-3ʹ
	R	5ʹ-TGTGCCTTGTCCCCGAAAAT-3ʹ
*STAT3*	F	5ʹ-CAGTTCTCGTCCACCACCAA-3′
	R	5ʹ-TCATTCCAAAGGGCCAAGAT-3ʹ
*CCND1*	F	5ʹ-CTCTCCTGCTACCGCACAACG-3ʹ
	R	5′-AGGCTTGACTCCAGAAGGGCT-3ʹ
*α-SMA*	F	5ʹ-CAGCTATGTGGGGGACGAAG-3ʹ
	R	5ʹ-TCCGTTAGCAAGGTCGGATG-3ʹ
*FSP-1*	F	5ʹ-ACCTCTCTGTTCAGCACTTCC-3ʹ
	R	5ʹ-GAACTTGTCACCCTCGTTGC-3ʹ
*COL I*	F	5ʹ-GAGAGGTGAACAAGGTCCCG-3ʹ
	R	5ʹ-AAACCTCTCTCGCCTCTTGC-3ʹ
*COL III*	F	5ʹ-AAGGCTGCAAGATGGATGCT-3ʹ
	R	5ʹ-GTGCTTACGTGGGACAGTCA-3ʹ
*GADPH*	F	5ʹ-TGGGAAGCTGGTCATCAAC-3ʹ
	R	5ʹ-GCATCACCCCATTTGATGTT-3ʹ

### Statistical analysis

Data were analyzed using SPSS 210 statistical software. Measurement information was expressed as mean ± standard deviation when it conformed to normal distribution and median (interquartile spacing) when it did not work to normal distribution. When analyzing the influencing factors, one-way ANOVA was used for between-group comparisons when the measures conformed to a normal distribution with a chi-square variance. If the difference was statistically significant, compare the data two by two using the Bonferroni test. The Kruskal–Wallis H-test was used to compare multiple groups when the information did not fit a normal distribution, or the variance was not homogeneous, and the Mann–Whitney U test was further used for two-by-two comparisons when the difference was statistically significant. A statistical difference was indicated by *P* < 0.05. Finally, use statistical graphs to create GraphPad Prism 9.5 software.

## Results

### Network pharmacology analysis

**Fig. 1:**
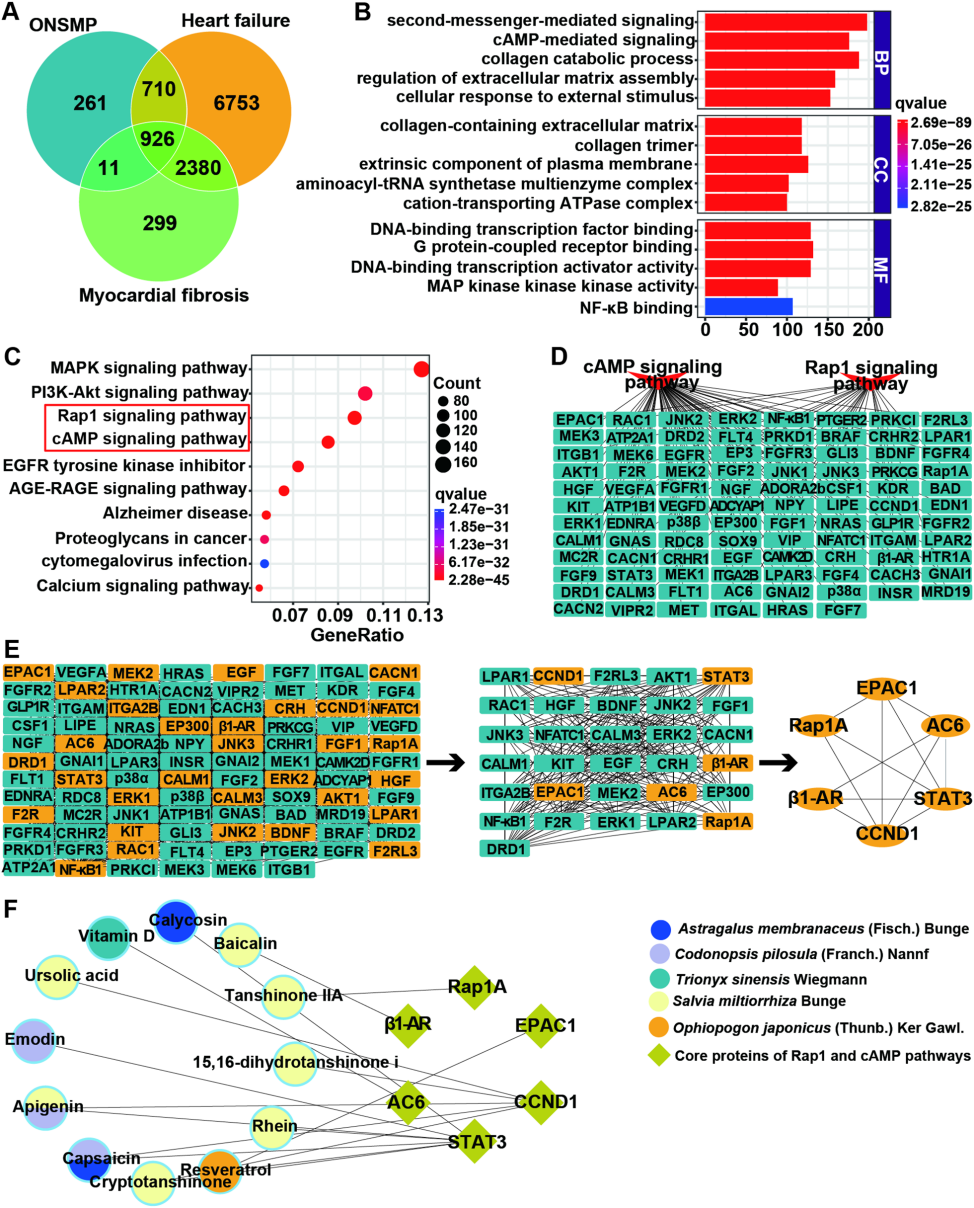
Network pharmacology analysis of ONSMP on myocardial fibrosis in heart failure. **A** Access to drug-disease intersection targets. **B** Bar graph of GO functional enrichment analysis of intersection targets. **C** Bubble diagram of KEGG signaling pathway enrichment analysis of intersection targets. **D** Topological analysis of the cAMP/Rap1A signaling pathway with enriched targets. **E** Acquisition of core targets of cAMP and Rap1A signaling pathways. The orange rectangle is the target that meets the screening conditions, the blue-green rectangle is the target that does not meet the screening conditions, and the orange oval is the core target. **F** Topological analysis of ONSMP active ingredients with core targets of cAMP and Rap1A signaling pathways

Through screening, we identified a total of 158 ONSMP active ingredients with good water solubility, intestinal absorption, and drug-like properties, targeting 1908 proteins (Additional file 1: Table S1). Meanwhile, we got 10,769 heart failure proteins and 3616 myocardial fibrosis proteins (Additional file 1: Table S2). Use Venn diagrams to plot intersecting proteins of ONSMP ingredients targets, heart failure proteins, and myocardial fibrosis proteins (Fig. 1A and Additional file 1: Table S3). The GO and KEGG enrichment analysis of intersecting proteins yielded 4812 functional entries and 216 signaling pathways. Among them, BP was 4149 entries, mainly involving collagen catabolic process, second-messenger-mediated signaling, and cAMP-mediated signaling; CC was 233 entries, primarily involving collagen-containing extracellular matrix, collagen trimer, and the extrinsic component of plasma membrane; MF was 430 entries, mainly involving DNA-binding transcription factor binding, G protein-coupled receptor binding, and DNA-binding transcription activator activity (Fig. 1B). The MAPK, PI3K-Akt, Rap1, and cAMP signaling pathways in the KEGG enrichment analysis results were closely associated with myocardial fibrosis in heart failure (Fig. 1C). The previous study of our group confirmed that ONSMP inhibits MAPK and PI3K-Akt signaling pathways to delay myocardial fibrosis. Therefore, the present study focused on exploring Rap1 and cAMP signaling pathways. There were 94 enriched proteins on the Rap1 and cAMP signaling pathways (Fig. 1D), and 6 core targets were screened (Fig. 1E). From the topological analysis of ONSMP components with core proteins, it is clear that β1-AR, AC6, EPAC1, and Rap1A can each be regulated by 1 ingredient, STAT3 by 7 ingredients, and CCND1 by 5 ingredients (Fig. 1F).

### Molecular docking analysis

**Fig. 2:**
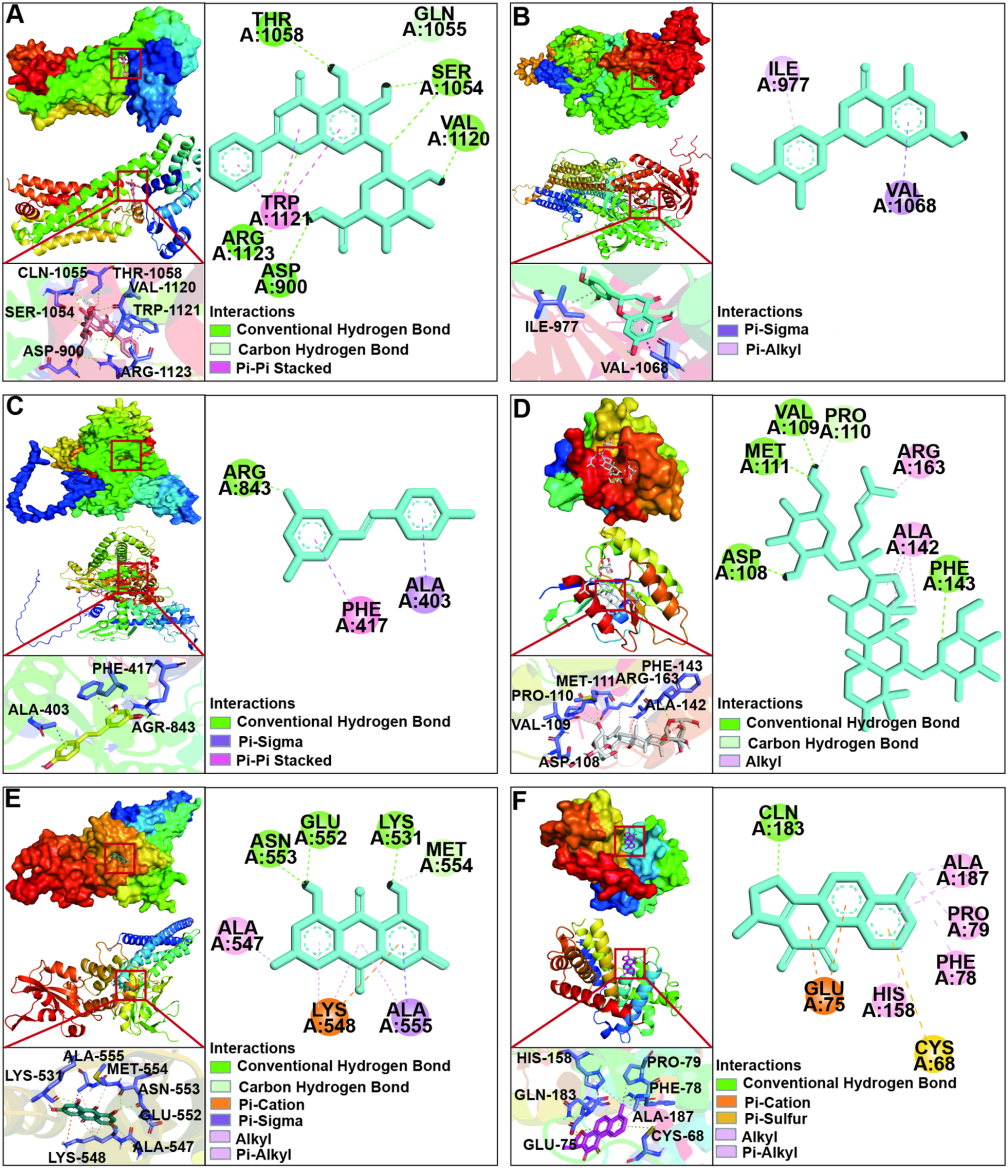
Action modes of active compounds with core targets. **A** Baicalin & β1-AR. **B** Vitamin D & AC6. **C** Resveratrol & EPAC1. **D** Tanshinone IIA & RAP1A. **E** Emodin & STAT3. **F** 15,16-dihydrotanshinone-i & CCND1

Generally speaking, affinity less than 0 kcal/mol indicates that the receptor and ligand bind spontaneously without external energy, affinity less than − 5 kcal/mol indicates excellent binding and less than − 7 kcal/mol indicates strong binding [19]. As shown in Table 4, the core targets and ONSMP active ingredients affinity were all less than − 5 kcal/mol. The core targets and ONSMP active ingredients with the best binding capacity were visualized and analyzed for interaction forces (Fig. 2). In the docking results, baicalin formed two carbon-hydrogen bonding interactions with amino acid residue GLN-1055 of the A-chain of the β1-AR protein, two carbon-hydrogen bonding interactions and three π-π conjugation interactions with TRP-1121, and seven conventional hydrogen-bonding interactions with ASP-900, SER-1054, THR-1059, VAL-1120, and ARG-1123; vitamin D forms a π-alkyl interaction with amino acid residue IL3-977 and a π-σ interaction with VAL-1068 in the A chain of the AC6 protein; resveratrol forms a π-σ interaction with amino acid residue ALA-403 of the A chain of the Epac1 protein, a π-π conjugation with PHE-417, and a conventional hydrogen bonding interaction with ARG-843; Tanshinone IIA formed four conventional hydrogen-bonding interactions with amino acid residues ASP-108, VAL-109, MET-111, and PHE-143 of the A chain of the Rap1A protein, one carbon-hydrogen-bonding interaction with PRO-110, and four alkyl interactions with ALA-142 and ARG-163; emodin formed three conventional hydrogen-bonding interactions with amino acid residues LYS-531, GLU-552, and ASN-553 of the A chain of the STAT3 protein, one carbon-hydrogen-bonding interaction with MET-554, one π-ionic-bonding interaction with LYS-548, one π-σ interaction with ALA-555, and four alkyl/π-alkyl interactions with ALA-547, LYS-548, and ALA-555; 15,16-dihydrotanshinone-I formed one π-sulfur bonding interaction with amino acid residue CYS-68 of the A-chain of the CCND1 protein, two π-ionic bonding interactions with GLU-75, one conventional hydrogen-bonding interaction with GLN-183, and five alkyl/π-alkyl interactions with PHE-78, PRO-79, HIS-158 and ALA-187. Indicating that the ONSMP active ingredients have the potential to affect the function and activity of the core proteins of the Rap1 and cAMP signaling pathways, thus achieving the purpose of treating diseases.

**Table 4:** Affinity of ONSMP active ingredients to core targets

Core target	Active component	Affinity
β1-AR	Baicalin	− 9.5
AC6	Vitamin D	− 7.7
EPAC1	Resveratrol	− 7.3
RAP1A	Tanshinone IIA	− 7.3
STAT3	Apigenin	− 6.1
	Calycosin	− 6.2
	Capsaicin	– 5.6
	Cryptotanshinone	− 6.1
	Emodin	− 7
	Resveratrol	− 5.8
	Rhein	− 6.8
CCND1	15,16-dihydrotanshinone-i	− 8
	Apigenin	− 7.2
	Capsaicin	− 6.6
	Cryptotanshinone	− 7.9
	Ursolic-acid	− 6.7

### Effect of ONSMP on cardiac function and ventricular structure

**Fig. 3:**
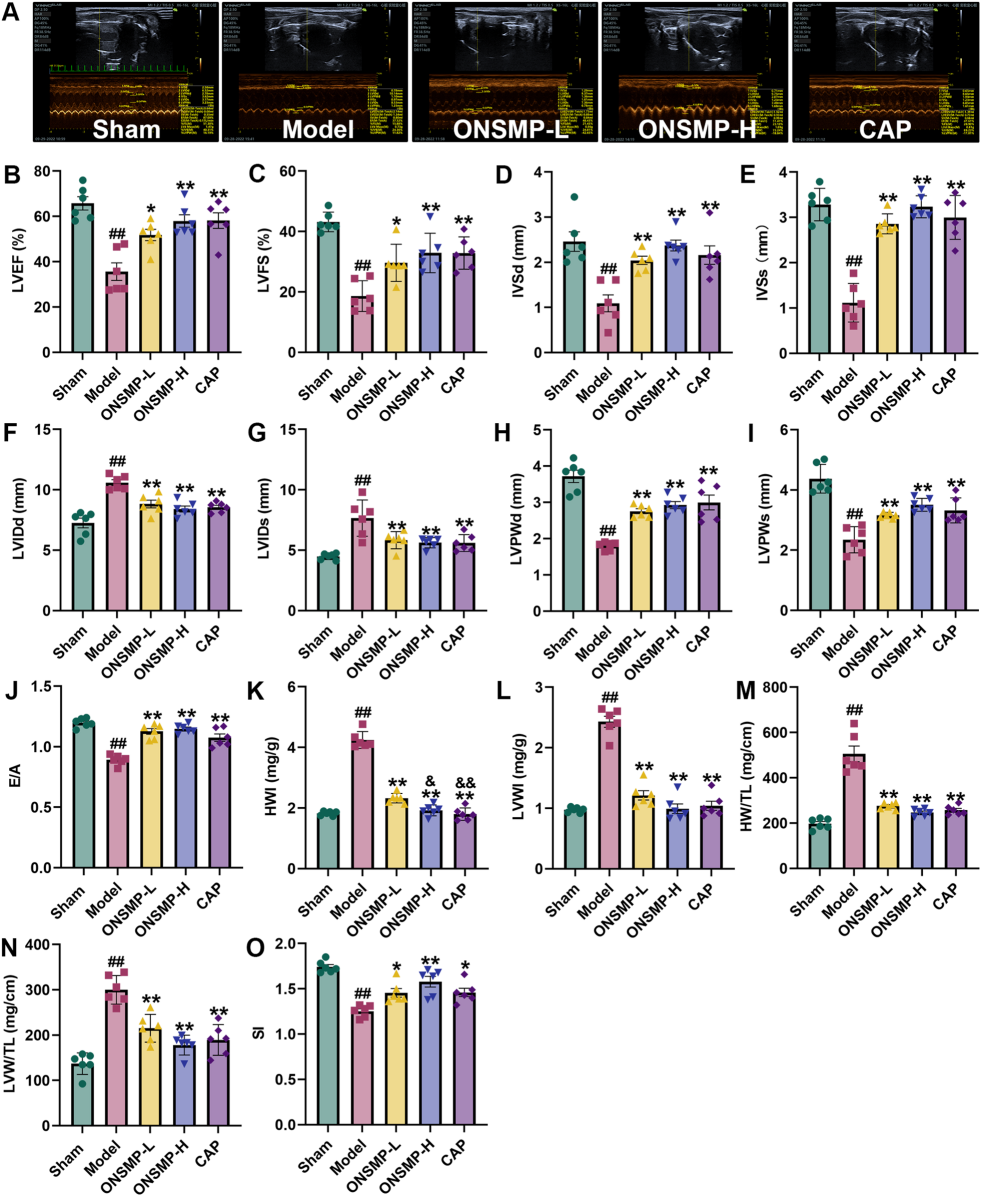
Echocardiographic and cardiac organ coefficient of rats in each group. **A** Typical echocardiograms. **B-O** Statistical plots of LVEF, LVFS, IVSd, IVSs, LVIDd, LVIDs, LVPWd, LVPWs, E/A, HWI, LVWI, HW/TL, LVW/TL, and SI. Data are presented as mean ± SD, n = 6. ^##^*P* < 0.01 vs the sham; ^*^P < 0.05, ^**^P < 0.01 vs the model; ^&^*P* < 0.05, ^&&^*P* < 0.01 vs the ONSMP-L

Echocardiography and cardiac organ coefficients can effectively reflect changes in rat heart function and ventricular structure. By echocardiography and cardiac organ, we found that LVEF, LVFS, LVSd, LVSs, LVPWd, LVPWs, E/A, and SI reduced, while LVIDd, LVIDs, HWI, LVWI, HW/TL, and LVW/TL were increased in rats in the model group compared to the sham group (*P* < 0.01). LVEF, LVFS, LVSd, LVSs, LVPWd, LVPWs, E/A, and SI increased, while LVIDd, LVIDs, HWI, LVWI, HW/TL, and LVW/TL decreased in each drug intervention group rats compared with the model group (*P* < 0.05 or 0.01). Compared to ONSMP-L, the HWI of rats in ONSMP-H and CAP groups reduced (*P* < 0.05 or 0.01) (Fig. 3).

### Effect of ONSMP on serum heart failure markers and myocardial cAMP

**Fig. 4:**
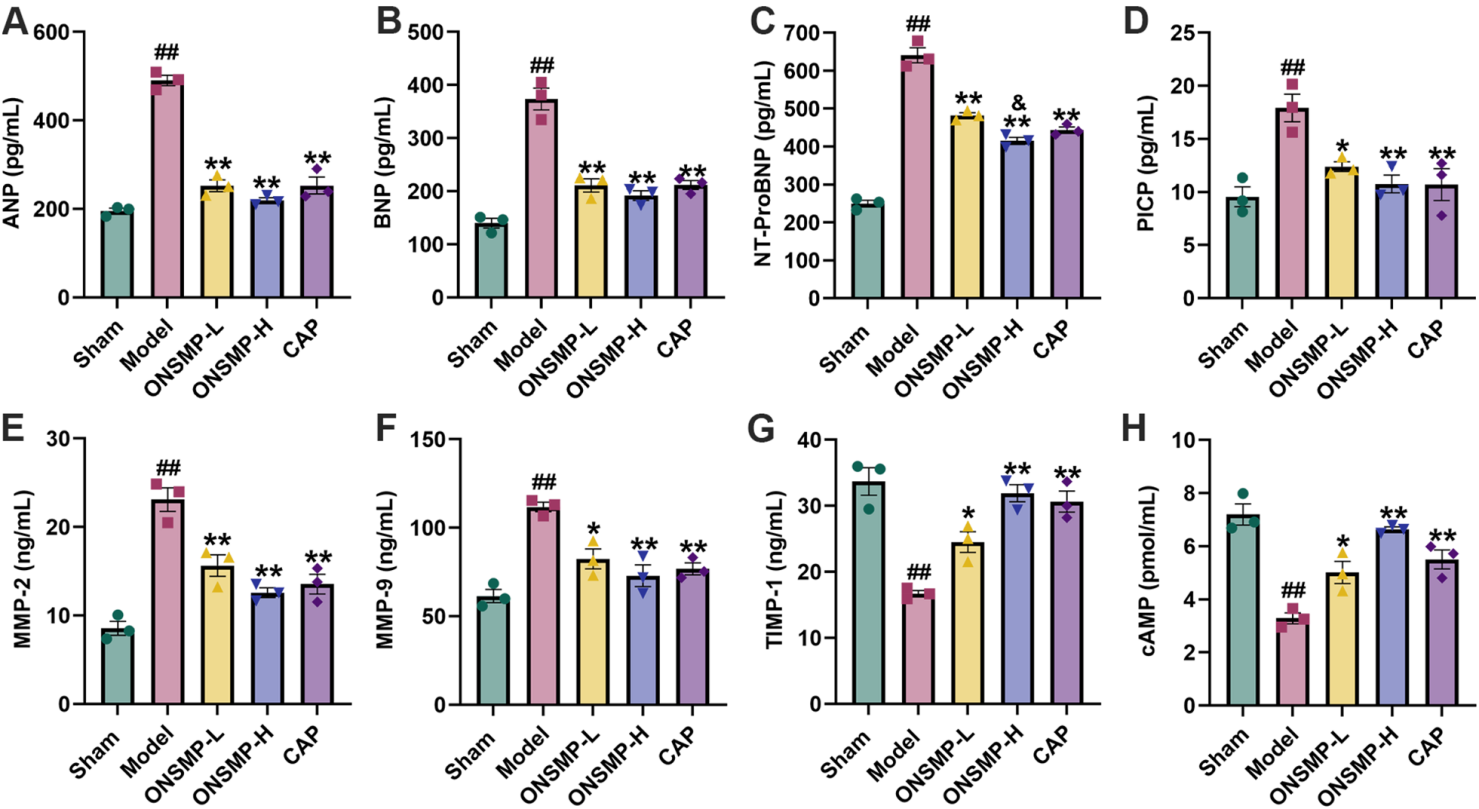
Serum heart failure markers and myocardial cAMP levels of rats in each group. **A**–**H** Statistical plots of ANP, BNP, NT-ProBNP, PICP, MMP-2, MMP-9, TIMP-1, and cAMP. Data are presented as mean ± SD, n = 3. ^##^*P* < 0.01 vs the sham; ^*^P < 0.05, ^**^P < 0.01 vs the model; ^&^*P* < 0.05 vs the ONSMP-L

The levels of ANP, BNP, NT-ProBNP, PICP, MMP-2, MMP-9, and TIMP-1 in rat serum reflect the severity of heart failure, and the level of cAMP in myocardial tissues can reflect the activity of the cAMP signaling pathway. Compared with the sham, ANP, BNP, NT-ProBNP, PICP, MMP-2, and MMP-9 levels increased, and TIMP-1 and cAMP levels decreased in model rats (*P* < 0.01). Compared with the model, ANP, BNP, NT-ProBNP, PICP, MMP-2, and MMP-9 levels decreased, and TIMP-1 and cAMP levels increased in each drug intervention group (*P* < 0.05 or 0.01). Compared with the ONSMP-L, NT-ProBNP level decreased in ONSMP-H group (*P* < 0.05). It indicated that ONSMP reduced the degree of heart failure in rats and had an activating effect on the cAMP signaling pathway (Fig. 4).

### ONSMP attenuates myocardial histopathologic changes and the positive expression of cardiac fibroblast markers

**Fig. 5:**
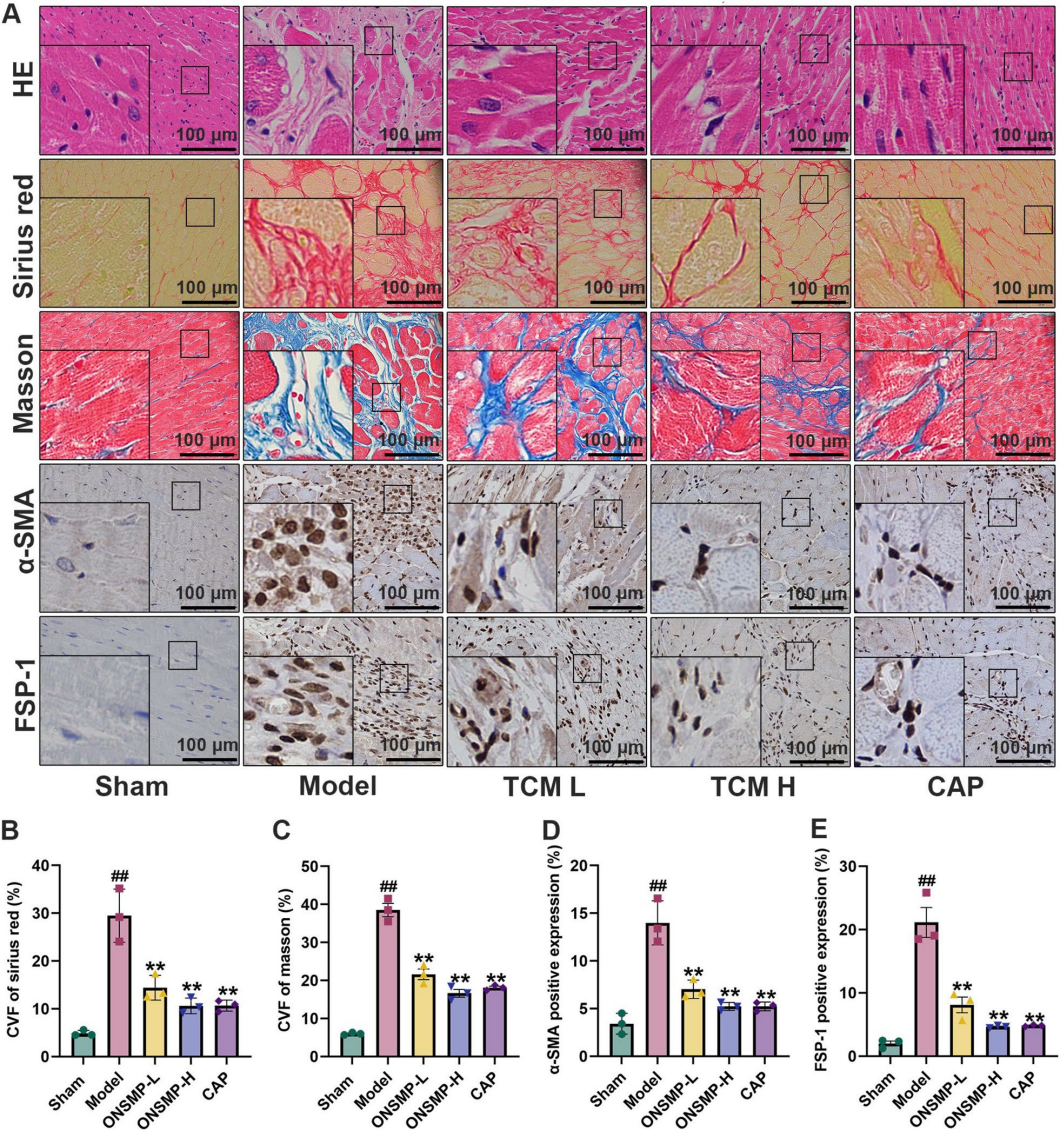
Myocardial histopathologic sections and immunohistochemical results of rats in each group. **A** Representative rat myocardial pathological sections (HE, Sirius red, and Masson) and immunohistochemical sections (α-SMA and FSP-1) (× 400). **B**–**E** Statistical plots of CVF of Sirius red, CVF of Masson, α-SMA positive expression, and FSP-1 positive expression. Data are presented as mean ± SD n = 3. ^##^*P* < 0.01 vs the sham; ^**^P < 0.01 vs the model

HE staining showed that myocardial fibers of sham were dense and well aligned, the number of cardiomyocytes was high, and the number of cardiac fibroblasts was meager; compared with sham, myocardial fibers of the model were fractured, disorganized, and poorly delineated, the number of cardiomyocytes reduced, and a large number of cardiac fibroblasts existed in the collagen fibers; myocardial injury and the alignment of myocardial fibers of the various drug intervention groups progressively improved when compared with the model (Fig. 5A). Sirius red staining showed that sham myocardial fibers were densely arranged, with only a few red collagen fibers in the interstitium; compared with sham, myocardial fibers in the model group were broken and disorganized, and the interstitium filled with red collagen fibers (*P* < 0.01); compared with the model group, collagen fibers of myocardial tissues of the rats in each administration group were gradually reduced (*P* < 0.01) (Fig. 5A and B). Masson staining showed that very few blue collagen fibers were visible in the interstitium of sham rat cardiomyocytes; compared with sham, the model group had a severe myocardial injury and a large number of blue collagen fibers in the interstitium of cardiomyocytes (*P* < 0.01); compared with the model, blue collagen fibers between rat cardiomyocytes were progressively reduced (*P* < 0.01) (Fig. 5A and C). IHC showed that the positive expression of α-SMA and FSP-1 (markers of cardiac fibroblasts) in the model group was significantly increased compared with the false-positive expression, suggesting an increase in cardiac fibroblasts (*P* < 0.01); the positive expression of α-SMA and FSP-1 in each administered group was gradually decreased compared with the model group (*P* < 0.01) (Fig. 5A, C and D).

### ONSMP regulates cAMP/Rap1A signaling pathway and myocardial fibrosis effector proteins

**Fig. 6:**
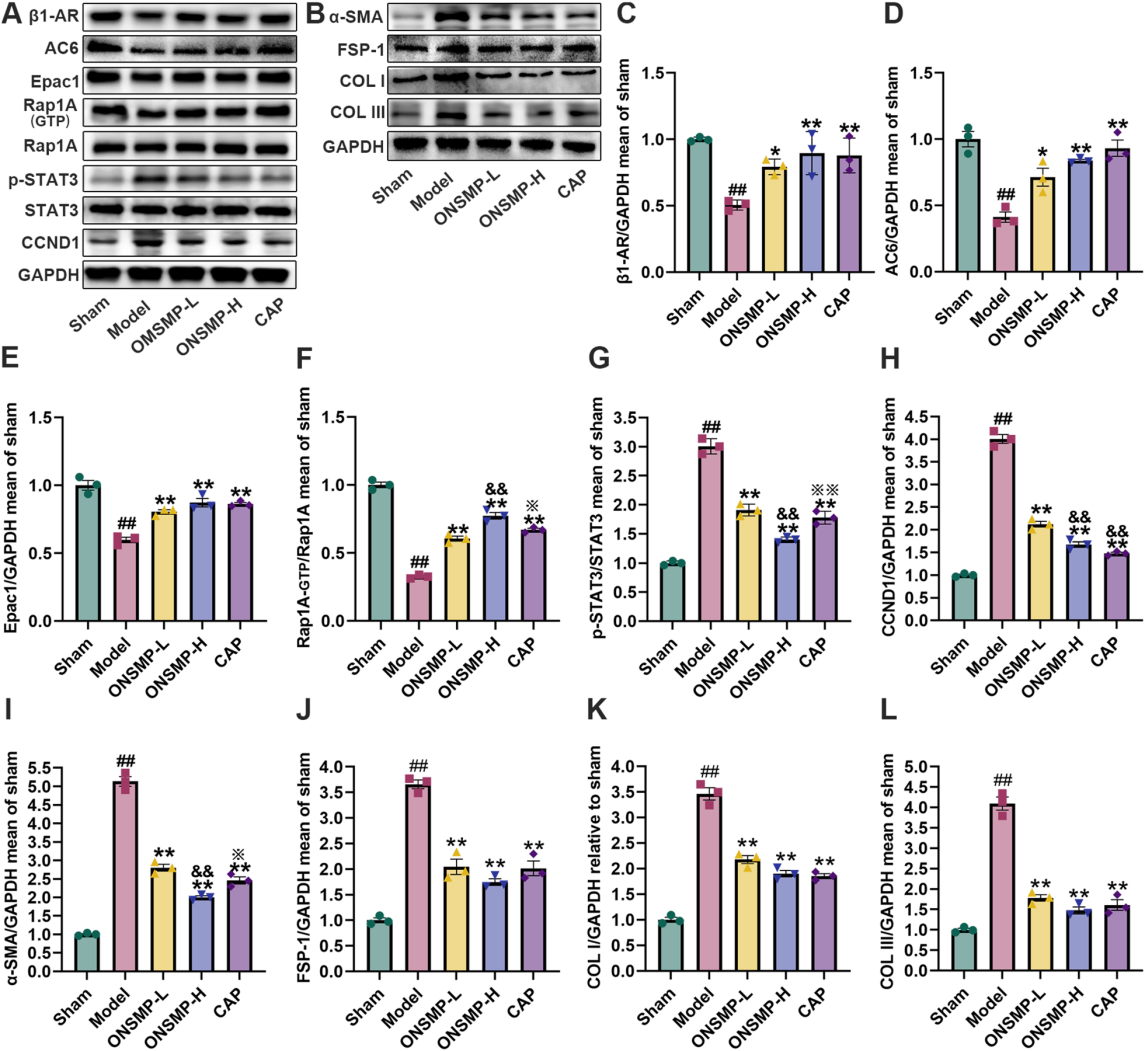
Effect of ONSMP on cAMP/Rap1A signaling pathway and myocardial fibrosis effector proteins. **A**, **B** Representative Western blot strips of cAMP/Rap1A signaling pathway and myocardial fibrosis effector proteins in myocardium with GAPDH as an internal reference. **C**–**L** Statistical plots of β1-AR, AC6, Epac1, Rap1A-GTP, p-STAT3, CCND1, α-SMA, FSP-1, COL I, and COL III. Data are presented as mean ± SD, n = 3. ^##^*P* < 0.01 vs the sham; ^*^P < 0.05, ^**^P < 0.01 vs the model; ^&&^*P* < 0.01 vs the ONSMP-L; ^※^*P* < 0.05, ^※※^*P* < 0.01 vs the ONSMP-H

By research, we found that β1-AR, AC6, Epac1, and Rap1A-GTP decreased, whereas p-STAT3, CCND1, α-SMA, FSP-1, COL I, and COL III elevated in the myocardium of the model rats compared with sham rats (*P* < 0.01). Compared with model rats, β1-AR, AC6, Epac1, and Rap1A-GTP were elevated in the myocardium of rats in each drug intervention group, whereas p-STAT3, CCND1, α-SMA, FSP-1, COL I, and COL III were decreased (*P* < 0.05 or 0.01). Compared with ONSMP-L, the CCND1 reduced in both ONSMP-H and CAP rat myocardium; a significant increase in Rap1A-GTP and a significant decrease in p-STAT3 and α-SMA were observed in the myocardial tissues of ONSMP-H rats (*P* < 0.01). p-STAT3 and α-SMA elevated in the myocardium of CAP rats compared with ONSMP-H rats (*P* < 0.05 or 0.01) (Fig. 6).

### ONSMP regulates cAMP/Rap1A signaling pathway and myocardial fibrosis effector mRNA

**Fig. 7:**
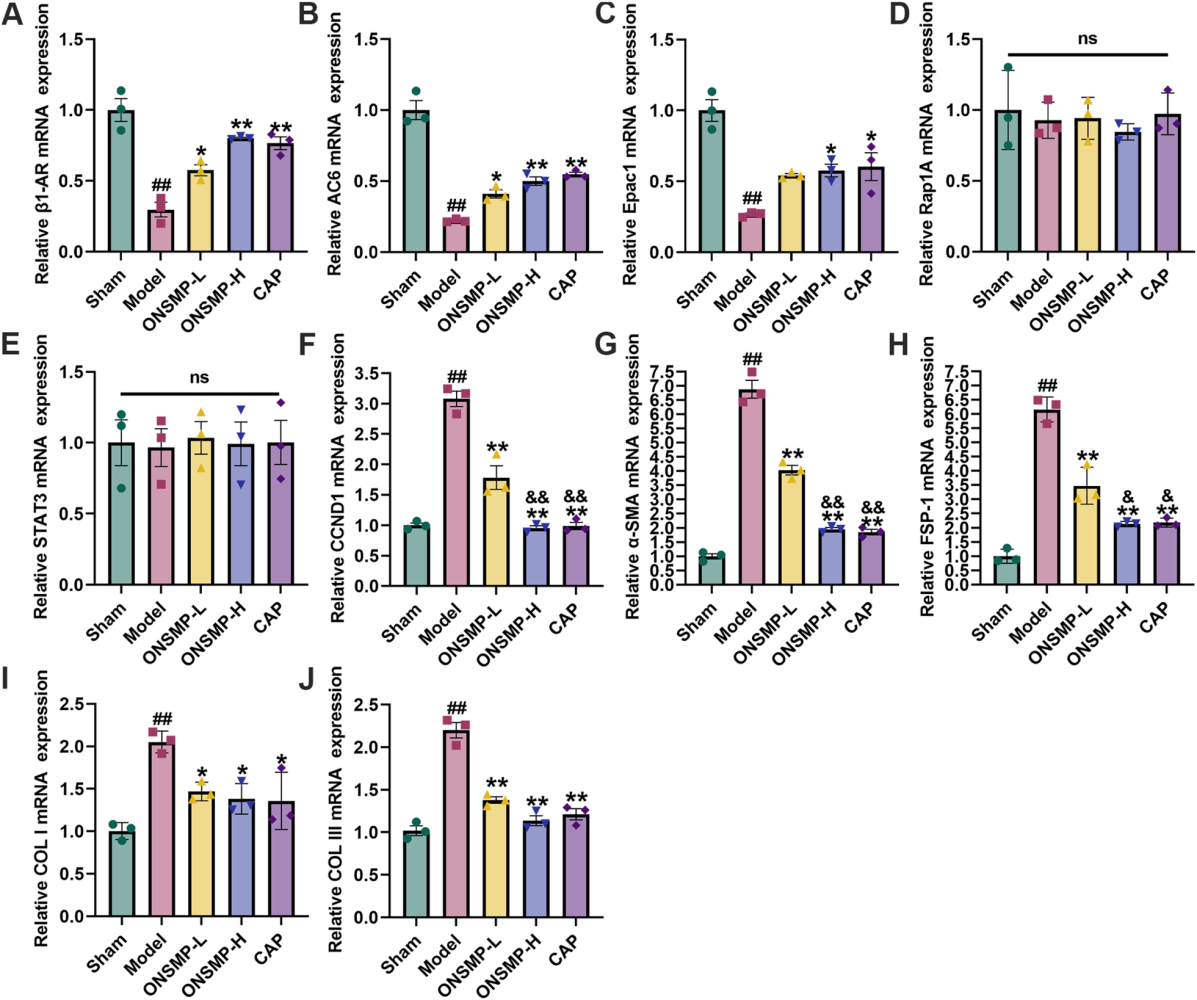
Effect of ONSMP on cAMP/Rap1A signaling pathway and myocardial fibrosis effector mRNA mRNA. **A**–**J** Statistical plots of β1-AR, AC6, Epac1, Rap1A, STAT3, CCND1, α-SMA, FSP-1, COL I, and COL III mRNA in myocardium. Data are presented as mean ± SD, n = 3. ^##^*P* < 0.01 vs the sham; ^*^P < 0.05, ^**^P < 0.01 vs the model; ^&&^*P* < 0.01 vs the ONSMP-L

By qPCR, we found no statistically significant differences in the mRNA levels of Rap1A and STAT3 in the myocardium of rats in all groups (*P* > 0.05). Compared with the sham, the mRNA levels of β1-AR, AC6, and Epac1 were decreased in the myocardium of model rats, whereas the mRNA levels of CCND1, α-SMA, FSP-1, COL I, and COL III were increased (*P* < 0.05 or 0.01). Compared with the model, mRNA levels of β1-AR and AC6 were increased, whereas mRNA levels of CCND1, α-SMA, FSP-1, COL I, and COL III decreased in each drug intervention group; mRNA levels of Epac1 increased in ONSMP-H and CAP (*P* < 0.05 or 0.01). The mRNA levels of CCND1, α-SMA, and FSP-1 were decreased compared with those of ONSMP-L (*P* < 0.05 or 0.01) (Fig. 7).

## Discussion

Heart failure is a significant challenge in the cardiovascular field in the twenty-first century, and how to inhibit myocardial fibrosis in multiple ways, to a greater extent, has become a research hotspot in treating heart failure [20]. Modern medicine believes that in the process of cardiovascular disease development, the continuous stimulation of ischemia, hypoxia, inflammation, and other pathological factors can seriously damage the myocardium and cause a large number of collagen fibers in the extracellular matrix of the myocardium, which restricts the pumping of blood in the heart [21]; traditional Chinese medicine believes that the pathomechanism of heart failure lies in the mutual aggravation of cardiac qi deficiency and blood stasis, phlegm, and dampness and other pathology products [22]. Clinical applications have gradually recognized the efficacy of ONSMP in treating heart failure. Still, at this stage, its specific active ingredients and molecular biological mechanisms have only been partially described, which needs to be more comprehensive. Therefore, the present study explored the signaling pathway, core targets, and active ingredients of ONSMP in treating myocardial fibrosis in heart failure more systematically and comprehensively by network pharmacology and verified them with molecular docking and animal experiments.

**Fig. 8:**
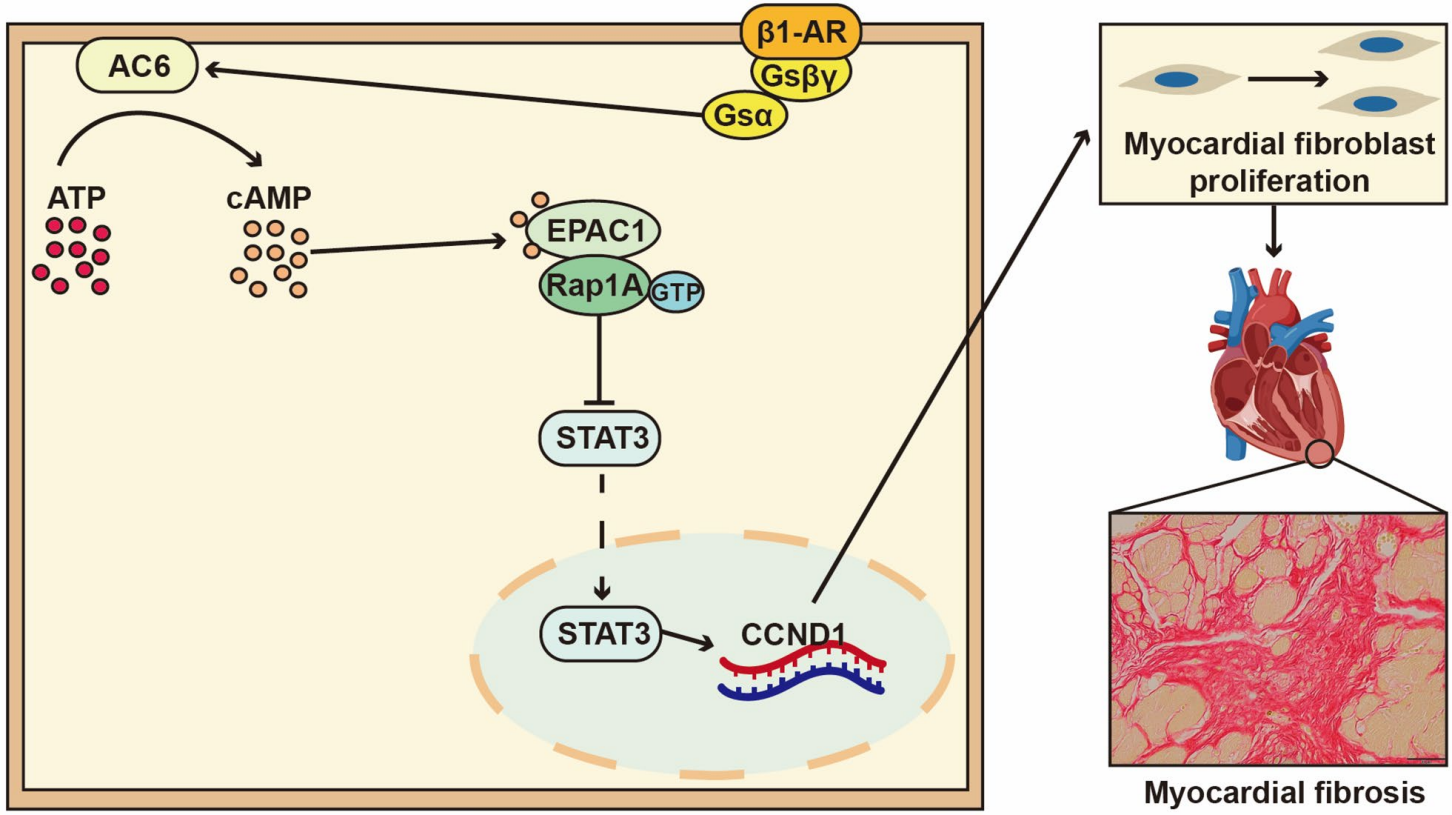
Relationship of the cAMP/Rap1A signaling pathway to myocardial fibrosis in heart failure

In the present study, based on the group's previous research, we found that the molecular biological mechanism associated with ONSMP inhibition of myocardial fibrosis in heart failure using a network pharmacology approach may also involve baicalin, vitamin D, resveratrol, tanshinone IIA, emodin, 15,16-dihydrotanshinone-i, and other active ingredients acting on β1-AR, AC6, EPAC1, RAP1A, STAT3, and CCND1 to regulate the cAMP/Rap1 signaling pathway. Scientific studies have found that the cAMP and Rap1 signaling pathways are interconnectable, and their signaling and possible mechanisms associated with myocardial fibrosis in heart failure are described below (Fig. 8): During the development of heart failure, there is a significant decrease in β1-AR on the cell membrane of cardiac fibroblasts [23–25], which affects the dissociation of the Gsα to which it is coupled, resulting in a significant decrease in the amount of AC6, the effector protein of Gsα, on the cell membrane [26]. Overexpression of AC6 in heart failure model mice has been reported to be effective in reducing myocardial infarct size, left ventricular remodeling, myocardial fibrosis, and mortality [27, 28]. Reduced expression of AC6 severely affects its ability to catalyze the generation of cAMP from ATP, resulting in lower cAMP levels [29]. Epac1 is a downstream target protein of cAMP [30], so in the absence of cAMP molecular interactions between Epac1’s own regulatory and catalytic domains inhibit its activity, thus preventing Epac1 from exposing the CDC25 structural domain from binding to its downstream effector Rap1A, resulting in reduced Rap1A activity [31]. Cai et al. demonstrated that knockdown of the Rap1A gene promotes STAT3 signaling [32]. Phosphorylated STAT3 into the nucleus can accelerate the transcription and expression of the CCND1 gene [33], which drives the cell cycle of cardiac fibroblasts from the G1 phase to the S phase and accelerates myocardial fibrosis [34].

Pharmacological studies have shown that baicalin can effectively inhibit myocardial fibrosis caused by various reasons by regulating peroxisome proliferators activated recepotor α/β/δ expression, p38 MAPK phosphorylation, AMPK/TGF-β/Smads signaling pathway, TGF β1/Smad2 signaling pathway, and other mechanisms [35–38]. Studies have shown that vitamin D attenuates fibrosis after myocardial infarction by inhibiting the TGF-β/Smads signaling pathway, and a combined metabolomics and transcriptomics analysis revealed that exogenous vitamin D supplementation also modulates AC6 [39, 40]. Resveratrol has a strong effect against cardiac fibrosis [41], and Park Sung-Jun et al. [42] found that resveratrol competitively inhibits phosphodiesterase degradation of cAMP and promotes Epac1 activation. Tanshinone IIA inhibits the expression of α-SMA protein, the NADPH oxidase activity, and oxidative stress involved in myocardial fibrosis [43–45]. Additionally, it was found that Tanshinone IIA regulates the RAP1 signaling pathway in the study of atherosclerosis [46]. The natural anthraquinone derivative emodin has a promising application as a therapeutic agent for cardiovascular diseases, and it also has a significant inhibitory effect on STAT3 activation [47–49]. 15,16-dihydrotanshinone-i is a natural abietane diterpenoid mainly found in the roots of Salvia miltiorrhiza Bunge, which induces cell cycle arrest in the G0/G1 phase by down-regulating CCND1 expression [50, 51].

Our findings are consistent with the modulatory effects of the corresponding components contained in ONSMP described above. We first constructed a rat model of heart failure by ligating the left anterior descending branch of the coronary artery, then performed the corresponding experiments at the molecular level of the cAMP/Rap1A signaling pathway to elucidate the mechanism of ONSMP in delaying myocardial fibrosis and provide an idea for the research of new drugs for heart failure treatment. The results of echocardiography, cardiac organ coefficients, serum heart failure markers Elisa assay, and histopathology showed that ONSMP differentially increased LVEF, LVFS, LVSd, LVSs, LVPWd, LVPWs, E/A, SI, and TIMP-1, and decreased LVIDd, LVIDs, HWI, LVWI, HW/TL, LVW/TL, ANP, BNP, NT-ProBNP, PICP, MMP-2, and MMP-9, attenuating the extent of myocardial injury, and reducing the size of myocardial fibrotic areas. Using Elisa for cAMP, immunohistochemistry for α-SMA and FSP-1, Western blotting, and qPCR for protein and mRNA expression of the cAMP/Rap1A signaling pathway and myocardial fibrosis effector, we found that ONSMP effectively up-regulated the cAMP content in rat myocardial tissues, decreased the α-SMA and FSP-1 positive expression, promoted the protein and mRNA expression of β1-AR, AC6, and Epac1, decreased the protein and mRNA expression of CCND1, α-SMA, FSP-1, COL I, and COL III, promoted the activation of Rap1A, and inhibited the activity of STAT3. It is thus clear that the mechanism by which ONSMP inhibits myocardial fibrosis in heart failure may be related to its regulation of the cAMP/Rap1A signaling pathway. By analyzing the differences between the ONSMP-L, H, and CAP groups in-depth, we found that in terms of pharmacodynamics, the modulation of cardiac function, cardiac organ coefficients, and levels of markers of heart failure was more pronounced with ONSMP-H than with ONSMP-L and that its effects were close to or slightly better than those of CAP. When investigating its mechanism of action, we found that ONSMP-H regulated the cAMP/Rap1A signaling pathway and the proteins and mRNAs of cardiac fibrosis effector molecules more significantly than ONSMP-L. In addition, ONSMP-H regulated cAMP, β1-AR, Rap1A, STAT3, α-SMA, and FSP-1 better than CAP. Considering that ONSMP is a traditional Chinese medicine compound with multi-pathway and multi-target characteristics, it is likely that its regulation of the cAMP/Rap1A signaling pathway also involves an indirect regulatory mechanism. In conclusion, ONSMP has a dose-dependent role in heart failure therapy and regulation of molecular mechanisms, and its modulation of the cAMP/Rap1A signaling pathway is superior to that of CAP. In summary, ONSMP has significant advantages in improving myocardial fibrosis in heart failure by regulating the cAMP/Rap1A signaling pathway.

Myocardial fibrosis is the pathologic basis of several cardiovascular diseases, which can ultimately lead to heart failure and an increased risk of death [52–54]. The main characteristic is excessive proliferation and differentiation of myocardial fibroblasts, which produce an extreme amount of collagen fibers [55, 56]. Myocardial fibrosis includes three categories: reactive myocardial fibrosis, replacement myocardial fibrosis, and infiltrative myocardial fibrosis. Reactive myocardial fibrosis is more common in hypertension [57], heart valve disease [58, 59], viral myocarditis [59, 60], and dilated cardiomyopathy [60]. The mechanism is increased pressure or volume load leading to an increase in collagen fibers surrounding the heart vessels but without myocardial cell loss or significant scarring [61]. Replacement myocardial fibrosis is more common in myocardial infarction, ischemia/reperfusion injury, and adriamycin cardiomyopathy. When large areas of cardiomyocytes are damaged or die, collagen fibers will repair the injury site and form fibrotic scars [62, 63]. Reactive and replacement myocardial fibrosis often coexist in different stages of the same disease. In the rat model of left ventricular overload, for instance, in the early stage of the disease, cardiomyocytes may enlarge but are not lost. The heart undergoes reactive myocardial fibrosis to maintain its structure and function. However, long-term pressure overload may cause partial necrosis and apoptosis of cardiomyocytes, leading to the development of replacement myocardial fibrosis [64]. The characteristic feature of infiltrative myocardial fibrosis is the accumulation of glycans and lipids in different types of cells in the heart, a phenomenon also seen in Fabry disease, a rare genetic disorder involving abnormalities in sphingolipid metabolism [65–67]. In this study, ONMP inhibited the proliferation of cardiac fibroblasts by regulating the cAMP/Rap1A signaling pathway. Cardiac fibroblasts are the most important effector cells of reactive and alternative myocardial fibrosis. Therefore, ONMP may have application value in treating heart failure caused by hypertension, valvular heart disease, viral myocarditis, dilated cardiomyopathy, myocardial infarction, ischemia/reperfusion injury, and adriamycin cardiomyopathy. However, the role of ONMP in infiltrative myocardial fibrosis still needs further exploration.

## Conclusions

This study shows that the possible mechanism of ONSMP in reducing myocardial fibrosis also includes the use of 12 active ingredients such as baicalin, vitamin D, resveratrol, tanshinone IIA, emodin, 15,16-dihydrotanshinone-i to regulate β1-AR, AC6, EPAC1, Rap1 A, STAT3, and CCND1 on the cAMP/RAP1A signaling pathway, thereby inhibiting the proliferation of cardiac fibroblasts and reduce the excessive secretion of collagen, effectively improve cardiac function and ventricular remodeling in rats with heart failure. However, the potential molecular biological mechanisms of ONSMP in the treatment of heart failure myocardial fibrosis are not limited to the results of the current stage of the study, and we believe that more in-depth studies will further reveal its many complex and sophisticated molecular biological mechanisms and provide a more scientifically adequate basis for the clinical application of ONSMP.

### Supplementary Information

**Additional file 1: Table S1.** ONSMP active ingredients and target proteins. **Table S2.** Disease proteins in heart failure and myocardial fibrosis. **Table S3.** Intersecting proteins of ONSMP component targets, heart failure proteins, and myocardial fibrosis proteins.

## Data Availability

The datasets used or analyzed throughout this study are available from the corresponding author upon reasonable request.

## Abbreviations

AC6: Adenylate cyclase 6
Akt: Protein Kinase B
ANP: Atrial natriuretic peptide
BNP: Brainnatriuretic peptide
BP: Biological process
cAMP: Cyclic adenosine monophosphate
CC: Cell component
CCND1: Cyclin D1
COL I: Collagen I
COL III: Collagen III
EPAC1: Exchange proteins activated by cAMP 1
FSP-1: Fibroblast-specific protein
GO: Gene ontology
Gsα: α-Subunit of the Gs protein
HW: Heart weight
HWI: Heart weight index
IVSd: Interventricular septal thickness at diastole
IVSs: Interventricular septal thickness at systole
LVEF: Left ventricular ejection fraction
LVFS: Left ventricular fraction shortening
LVIDd: Left ventricular end diastolic dimension
LVIDs: Left ventricular end-systolic dimension
LVPWd: Left ventricular posterior wall diastolic thickness
LVPWs: Left ventricular posterior wall systolic thickness
LVW: Left ventricular weight
LVWI: Left ventricular weight index
MAPK: Microtubule-associated protein kinase
MF: Molecular function
MMP-2: Matrix metalloproteinase 2
MMP-9: Matrix metalloproteinase 9
NT-ProBNP: N-terminal pro-B type natriuretic peptide
PICP: Carboxyterminal propeptide of type I procollagen
PI3K: Phosphatidylinositol 3-kinase
PVDF: Polyvinylidene difluoride
qPCR: Quantitative real-time PCR
Rap1A: RAS-related protein-1a precursor
RalGDS: Ral guanine nucleotide dissociation stimulator
SI: Sphericity index
STAT3: Signal transducer and activator of transcription 3
TIMP-1: Tissue inhibitor of metalloproteinases-1
TL: Tibial length
α-SMA: α-Smooth muscle actin
β1-AR: β1 Adrenergic receptor
